# Molecular characterization of retinitis pigmentosa in Saudi Arabia

**Published:** 2009-11-24

**Authors:** Mohammed A. Aldahmesh, Leen Abu Safieh, Hisham Alkuraya, Ali Al-Rajhi, Hanan Shamseldin, Mais Hashem, Fatemah Alzahrani, Arif O. Khan, Faisal Alqahtani, Zuhair Rahbeeni, Mohammed Alowain, Hanif Khalak, Salwa Al-Hazzaa, Brian F. Meyer, Fowzan S. Alkuraya

**Affiliations:** Department of Genetics, King Faisal Specialist Hospital and Research Center, Riyadh, Saudi Arabia

## Abstract

**Purpose:**

To catalog mutations that underlie retinitis pigmentosa (RP) in Saudi Arabia using a representative sample.

**Methods:**

Fifty-two patients with RP were recruited and their homozygosity mapping, with or without linkage analysis, was used to suggest the causative genes followed by bidirectional sequencing.

**Results:**

Mutations were identified in 94% of our study cohort, including seven that were novel.

**Conclusions:**

Homozygosity mapping is an extremely robust approach in the study of retinitis pigmentosa in the setting of high rates of consanguinity. *BBS3* mutations can rarely present as nonsyndromic RP.

## Introduction

The retina is an elaborate eye structure that is responsible for transforming light energy into chemical and electrical energy, which the brain decodes to produce normal visual perception [1]. Photoreceptors play a key role in this complex process, and functional or structural defects in these specialized receptors adversely affect retinal function. The nonspecific term “retinal dystrophy” has long been used as a clinical descriptor of hereditary conditions characterized by progressive loss of photoreceptors associated with an abnormal appearance of the retina [2]. Some of these conditions appear specifically to affect the photoreceptors concerned with color vision (cones), while others are characterized by more significant involvement of dark vision photoreceptors (rods). Still others affect photoreceptors indirectly by causing progressive damage to the supporting retinal pigment epithelium [3]. Retinitis pigmentosa (RP) represents the most common form of retinal dystrophies, and while it preferentially affects rod function, cone function is also affected in advanced cases [4]. The age of onset is also variable, with some patients presenting at birth (this is usually referred to as Leber congenital amaurosis, or LCA) and others presenting later in childhood or even in adulthood [5]. With more than 51 genes and loci known to be involved in the pathogenesis of RP, the delineation of the underlying genetic defect represents a major challenge in establishing the molecular diagnosis for these patients. However, identifying the mutation is of great clinical utility because it is a prerequisite to key preventive measures such as prenatal diagnosis and preimplantation genetic diagnosis. Furthermore, with the recent advances in gene therapy for some cases of RP [6,7], there is increasing interest among patients to know their mutation, since it might determine their eligibility for ongoing and future clinical trials relevant to their specific gene mutation.

While genetic defects that cause RP are known to follow all modes of Mendelian inheritance, autosomal recessive forms of RP are estimated to contribute 50%–60% to the overall mutation pool of the disease [8]. Consanguinity is known to increase the frequency of recessive disorders since it renders part of the genome homozygous by descent, which increases the likelihood of the appearance of recessive ancestral mutations in the homozygous state [9,10]. We hypothesized that autosomal recessive RP will account for the majority of cases in the highly consanguineous Saudi population. Consequently, mapping of blocks of homozygosity in RP patients from Saudi Arabia could serve as a shortcut to the identification of the likely candidate genes, as an alternative to the massive sequencing effort that is usually needed to make the molecular diagnosis in patients with this genetically heterogeneous disorder. In this study, the largest to date on Saudi RP patients, we show that homozygosity mapping is indeed a powerful tool for the rapid and efficient identification of the causative mutations of this condition.

## Methods

### Human subjects

Patients with RP were identified using established ophthalmological criteria that are based on the gross appearance of the retina and on a detailed functional assessment [11]. With the exception of patients with syndromic forms, all referred patients with RP were enrolled in this study regardless of the age of onset. A written consent form was signed by all participants and/or their legal guardians. Thorough family histories were obtained from all patients, who were accordingly categorized as either sporadic or familial. Patients were recruited through King Khaled Eye Specialist Hospital and King Faisal Specialist Hospital and Research Center. This study was approved by the Institutional Review Board at King Faisal Specialist Hospital and Research Center (RAC#2070023) in accordance with the Declaration of Helsinki.

### DNA and RNA extraction

DNA was extracted from whole blood using standard protocol. Blood was also collected for RNA extraction in PAXGene tubes (Qiagen, Germantown, MD), whenever possible, from all sporadic cases and from at least the index in familial cases.

### Genotyping

Genome-wide genotypes were obtained using an Affymetrix SNP 250K Chip platform (Affymetrix, Santa Clara, CA) following the manufacturer’s instructions. Blocks of homozygosity were identified using either the Affymetrix® Genotyping Console™ (Affymetrix) or Copy Number Analyzer for GeneChip® arrays, Version 3.0 (CNAG, Tokyo, Japan) [12].

### Linkage analysis

When three or more affected individuals were identified in one family, linkage analysis was performed on the SNP genotypes using the Allegro component of EasyLinkage software [13].

### Mutation analysis

Genes suggested by linkage analysis and/or homozygosity mapping were PCR-amplified using primers that covered the entire coding sequence, as well as the flanking intronic sequences. PCR amplification was performed on a thermocycler (DNA Engine Tetrad. MJ Research, Inc., Waltham, MA) in a total volume of 25 µl. PCR primers as well as reaction conditions are available upon request. PCR amplicons were submitted for bidirectional sequencing using an Amersham ET Dye Terminator Cycle Sequencing Kit (Amersham Biosciences, Piscataway, NJ) following the manufacturer’s instructions. Sequence analysis was performed using the SeqManII module of the Lasergene (DNA Star Inc., Madison, WI) software package, with a normal sequence used for comparison. All missense mutations were confirmed by screening at least 100 Saudi control individuals and by checking for conservation across different species.

## Results

### Human subjects

We were able to enroll 52 patients representing 6 sporadic and 11 familial cases. Only three patients (one family) had a family history suggestive of dominant inheritance, and no patients appeared from their histories to have the X-linked form of RP. Table 1 summarizes the families enrolled and their phenotypes (LCA versus RP with onset later in life).

**Table 1:** Summary of gene mutations.

Family ID	N	Phenotype	Gene	Mutation	Homozygous versus Heterozygous	Effect of Mutation	No. of reported mutations in the literature	Reference	GenBank accession number
DGU-F3-DGU-F10	27	LCA	*TULP1*	c.895C>T; p.Q301X	Homozygous	Premature truncation	23	[16]	NM_003322.3
DGU-F13	3	LCA	*CRX*	c.458delC; p.P153fsX	Heterozygous	Frameshift and premature truncation	39	[20]	NM_000554.3
DGU-F14	3	LCA	*RDH12*	c.226G>C; p.G76R	Homozygous	Replacement of a highly conserved glycine residue by arginine	31	this study	NM_152443.1
DGU-F1	3	RP	*RP1*	c.662delC; p.A221GfsX20	Homozygous	Frameshift and premature truncation	48	this study	NM_006269.1
DGU-F2	3	RP	*RP1*	c.606C>A;p.D202E	Homozygous	Replacement of a highly conserved aspartic acid residue in the 2nd Doublecortin domain	this study		
DGU-F11	4	RP	*CRB1*	c.3159T>G; p.C1053W	Homozygous	Replacement of a highly conserved threonine residue in the 3rd Laminin G-like domain	102	this study	NM_201253.1
DGU-F12	1	RP	*CRB1*	c.80G>C; p.C27F	Homozygous	Replacement of a highly conserved cysteine residue in the 2nd EGF-like domain	this study		
DGU-F15	4	RP	*ARL6 *(BBS3)	c.619C>T; p.A89V	Homozygous	Replacement of a highly conserved alanine residue by valine	0	this study	NM_032146.3
DGU-F16	3	RP	*MERTK*	c.1335delAG; R445RfsX28	Homozygous	Frameshift and premature truncation	11	this study	NM_006343.2
DGU-F17	1	RP	N/A	N/A	N/A	N/A	N/A	N/A	N/A

Abbreviations: LCA represents Leber congenital amaurosis, RP represents retinitis pigmentosa, Homo represents Homozygote, Hetero represents Heterozygote, N/A represents Not applicable.

### Linkage analysis

Eight families had three or more affected members, and were deemed suitable for traditional linkage analysis. Family DGU-F13 was suggestive of an autosomal-dominant pattern of inheritance. Despite the limited number of meiotic recombinants (three affected and four unaffected in two generations), we were able to generate a few linkage peaks, one of which overlapped with the *CRX1* locus. Of the remaining seven families, only two (DGU-F4 and DGU-F5) gave a definitive single linkage peak with a significant LOD score (>3.5) corresponding to the *TULP1* locus. In contrast, in the remaining five families, multiple linkage peaks were obtained (averaging three peaks for LOD scores of 2–3), and subsequent analysis was only possible with the aid of CNAG (see below). In all these families, mutation analysis confirmed the presence of pathogenic mutations in the linkage loci (see below). Surprisingly, Family DGU-F15 was found to link to the *BBS3* locus even though the phenotype was clearly that of nonsyndromic retinitis pigmentosa.

### Homozygosity mapping

After the exclusion of Family DGU-F13 (which was autosomal dominant), Families DGU-F4 and F5 (for which definitive one-linkage peaks were obtained), and the 10 patients for whom targeted mutation analysis was positive (see below), all remaining patients (n=25) had their DNA genotyped to determine their blocks of homozygosity. Consistent with the fact that 94% of these patients came from consanguineous families, multiple blocks of homozygosity were usually seen for each patient (averaging 3.4). These averaged 33 Mb in size, ranging from 5 to 40 Mb. With the exception of one sporadic case (DGU-F17), at least one of these regions overlapped with a locus known to be involved in RP.

### Mutation analysis

**Figure 1:**
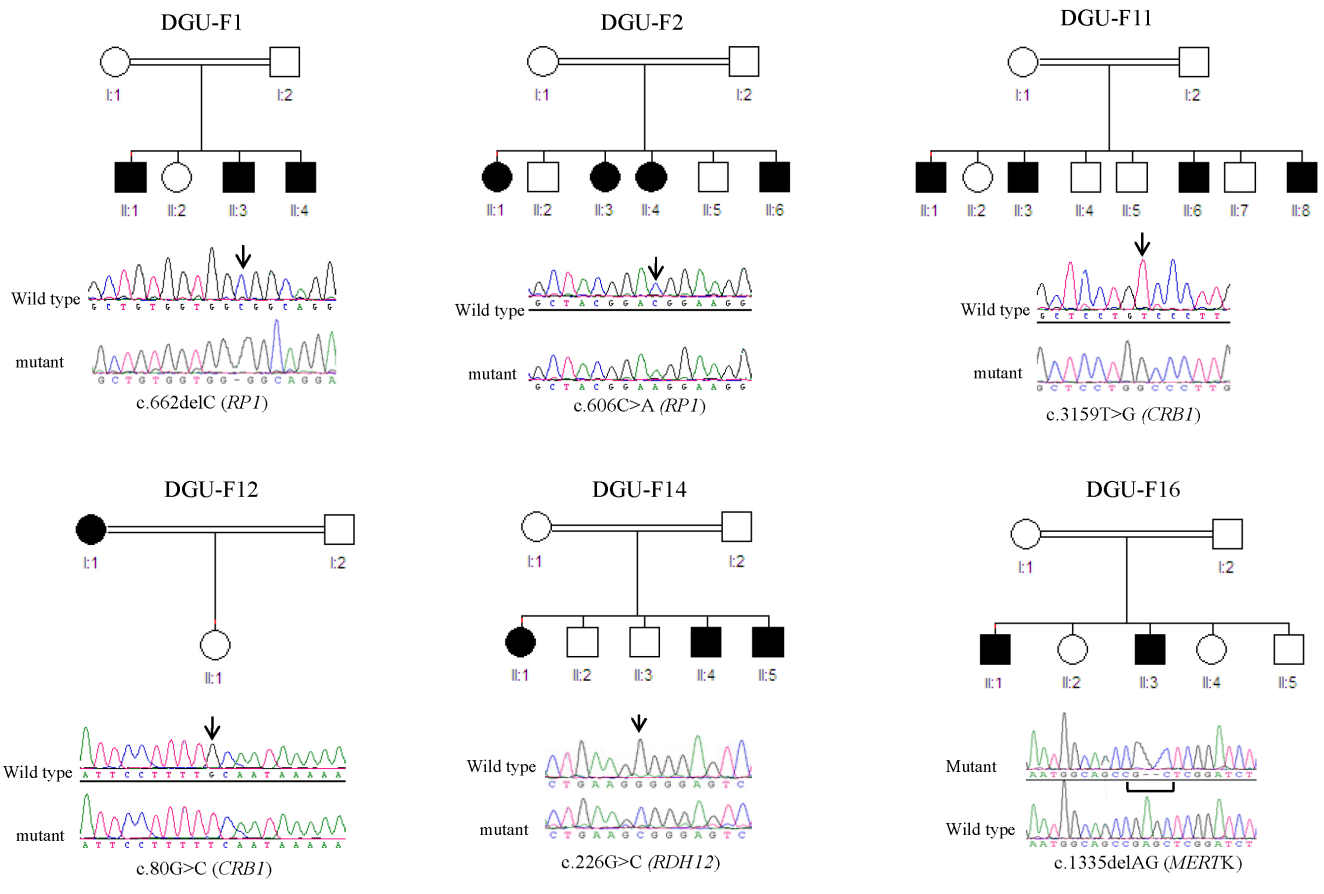
Summary of the novel mutations identified in this study. Simplified pedigrees are shown for each of the families (circle for female, square for male, white for unaffected, and black for affected). Below each pedigree, a sequence chromatogram is shown for the corresponding mutation, with a wildtype tracing for comparison (arrow indicates the position of the mutation on the chromatogram).

**Figure 2:**
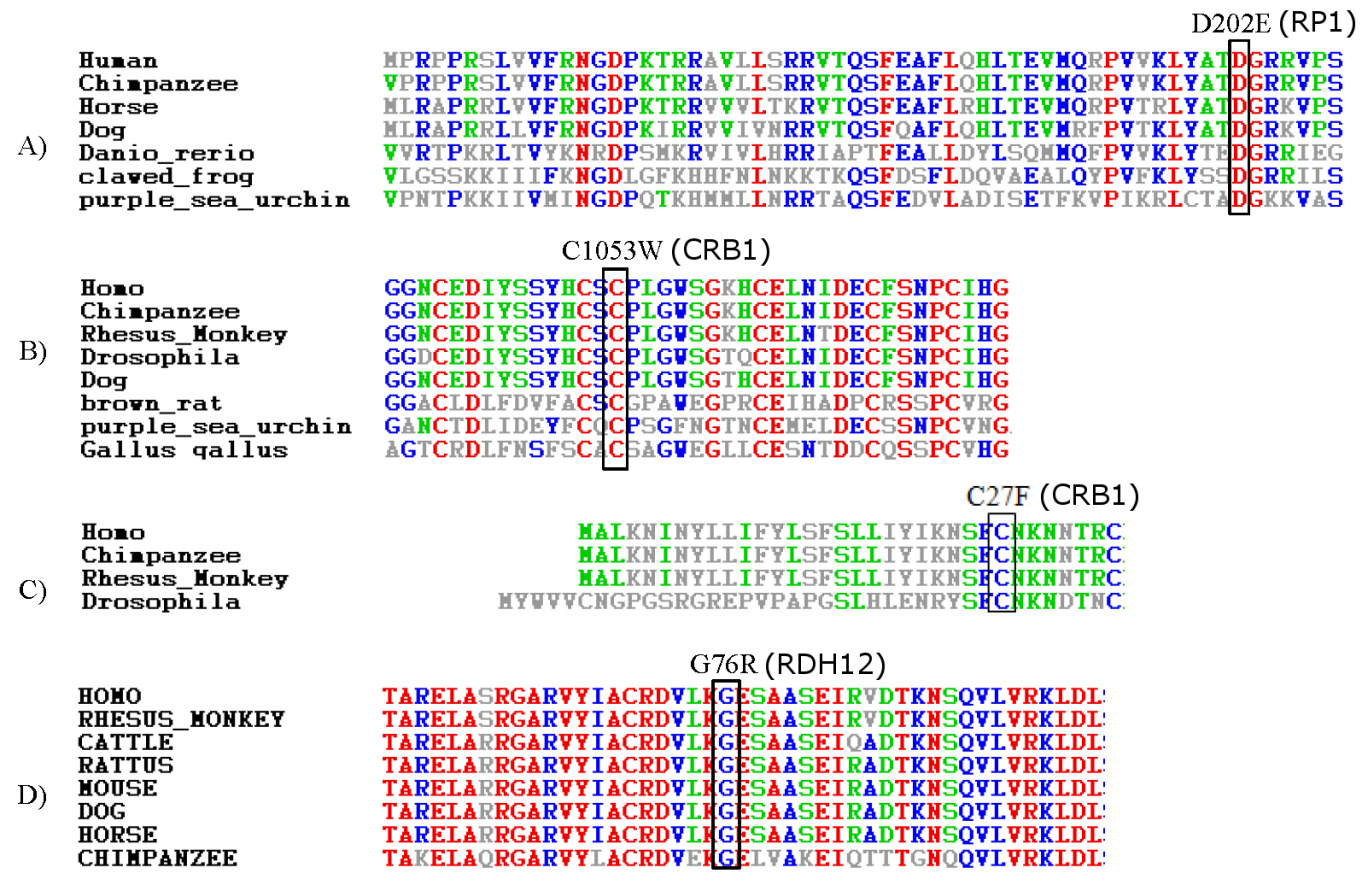
Analysis of the conservation level of the missense mutations identified in this study. For each missense mutation, a panel of orthologs from different organisms is shown to demonstrate the conservation of the involved residue across species, which suggests that a change of that residue may adversely affect the protein function.

Table 1 summarizes all mutations identified in the course of this study. In all, we have identified the causative mutation in 51 out of our 52 patients, and seven of these mutations were novel (Figure 1). None of the novel missense mutations was found in a panel of at least 100 ethnically matched controls (≥200 chromosomes), and all were found to fully segregate with the disease, whenever applicable i.e., in families with more than one affected member. Furthermore, strong conservation across species was demonstrated for all of them (Figure 2).

Linkage to *TULP1,* which was identified by linkage analysis in Families DGU-F4 and F5 (14 patients) was followed up by mutation analysis, which confirmed the presence of a pathogenic mutation (Figure 1). Haplotype analysis confirmed the ancestral nature of this mutation. This was the first mutation identified in this study so to examine the contribution of this founder mutation to the overall mutation pool, we have undertaken targeted mutation analysis of all of our samples (except for Family DGU-F13 with autosomal dominant inheritance). This approach allowed us to identify 10 additional patients representing five families (DGU-F3, F6, F7, F8, F9, and F10). For all the remaining patients (n=25), we systemically screened for mutations in known RP genes that reside in blocks of homozygosity. The number of genes suggested by our homozygosity mapping ranged from 1 to 10 per patient. The average number of genes sequenced per patient was two. Pathological mutations were identified in all but one patient (Table 1).

Notably, our list of novel mutations included one in *MERTK*, one of the least frequent causes of RP [14,15]. The highly surprising finding of a linkage in Family DGU-F15 was pursued with mutation analysis of *BBS3,* which revealed a novel missense mutation. Based on this finding, the family was called back for a comprehensive clinical genetics evaluation, which confirmed the complete lack of any apparent or visceral manifestation of Bardet-Biedl syndrome other than retinitis pigmentosa (unpublished).

## Discussion

Recently, a study on the genetics of Leber congenital amaurosis in 38 Saudi families [16] reported 24% mutation detection. While our study cohort was smaller (17 families), we believe the remarkably higher mutation detection rate found in the current study can be attributed to the difference in methodology. Li and colleagues selectively screened for mutations in 13 known LCA genes by direct sequencing. Our approach, on the other hand, was not biased toward a particular group of genes. The very large network of referring physicians from all parts of the country, as well as the largest specialized eye hospital (King Khaled Eye Specialist Hospital) makes it likely that our cohort was a representative sample of the RP population in Saudi Arabia. Our group has previously shown how consanguinity can override founder effect and allow for greater genetic and allelic heterogeneity than expected for populations characterized historically by limited mixing of their genetic pool, as is the case for the population of Saudi Arabia [17]. Indeed, the results of our analysis show that with the exception of the *TULP1* mutation, our nine mutations, seven of which were novel, are private mutations that are only seen in isolated familial or sporadic cases. Of note is that the phenotype associated with the novel mutations (LCA versus RP with onset later in life) is consistent with previously published mutations in those genes. Remarkably, of the 16 families for whom a mutation was assigned, 15 (93.75%) had autosomal recessive mutations that explained their condition. This extraordinary contribution from the autosomal recessive category to the overall disease pool was remarkably higher than what has previously been published on the genetics of this condition [8]. This is consistent with the fact that 92% of our patients come from consanguineous families, a much higher percentage than the national average of 56% [18]. This should not be viewed as a consequence of the bias of our homozygosity approach, which can only aid in the identification of recessively acting mutations. Indeed, this percentage was calculated based on all patients (n=52) who were enrolled regardless of the apparent mode of inheritance. One may argue that patients with recessive mutations are more likely to be referred, compared to patients with de novo dominant mutations as a result of their more conspicuous familial clustering. Indeed, only 11% of our study’s patients with recessive mutations were sporadic and without a family history. However, the latter finding is of particular relevance to the design of testing algorithms in the future, since it suggests that even in the absence of family history, Saudi RP patients are still likely to have the autosomal recessive form of the disease. This makes homozygosity mapping a particularly appealing approach, as it is very likely to aid in molecular diagnosis in the overwhelming majority of Saudi RP patients. We believe that the clinical utility of this approach is currently superior to that of other strategies. For example, the prioritization of genetic testing based on frequency data are still not applicable in Saudi Arabia, since such data do not exist yet. Indeed, this study, given its size, falls short of addressing this, and more studies will need to be conducted in the future, to more comprehensively catalog the mutational spectrum of this condition in Saudi Arabia. For the same reason, the targeted mutation screening approach is not very practical at the moment. Additionally, as we have shown in previous work [17], we expect the majority of mutations to be private mutations, which limits their generalizability. Very recently, Bergen et al. [19] have suggested the use of a sequencing chip that sequences genes known to cause retinal dystrophy. While this approach is probably very practical in outbred populations, we believe that, in highly consanguineous populations, homozygosity mapping offers significant cost savings over this approach that involves the routine generation of 400 PCR amplicons for each patient sample [19].

In summary, our study represents the largest molecular study of RP (both LCA and delayed onset) in Saudi Arabia to date. Autosomal recessive forms account for the overwhelming majority of RP in our population, which particularly suits it for homozygosity mapping, a powerful, high-throughput approach with significant savings in time and cost. Our analysis adds seven novel mutations to the list of mutations in the known RP genes, including the highly unusual mutation in *BBS3* in a patient with nonsyndromic retinitis pigmentosa. The clinical utility of this study will be further enhanced by our ongoing effort to recruit more families for molecular testing.
